# The Annual Meeting of the Medical Association of Georgia

**Published:** 1891-05

**Authors:** 


### Editorial.

### THE ANNUAL MEETING OF THE MEDICAL ASSOCIATION OF GEORGIA.

To the few members of the Medical Association of Georgia who are in the habit of regularly attending its meetings year after year, a striking feature lies in the almost complete change in the *personnel* of these gatherings, as they successively occur in different parts of the State. The members who were present in Rome, and who made that meeting one which will long be remembered in the history of the Association, were conspicuous chiefly by their absence at the meetings in Macon and Brunswick, while those who contributed to the eminent success of the Association when it met in the latter cities were sought in vain last month in Augusta. This fact has been urged as a reason for the permanent location of the Association in one of the central cities of the State, Atlanta or Macon. But to our mind it furnishes an argument against the adoption of such a course. It is the policy of the Association, or should be, to bring within its sheltering arms every regular physician in the State. A permanent location in one city would exclude from attendance, if not from membership, many practitioners in remote sections who would not wish to make the sacrifice involved in a long journey. Membership would gradually become limited to residents of the vicinity of the place of meeting until the Association would finally be practically converted into a local organization. That this danger is not a fanciful one is shown by the well-known history of the old Georgia Medical Society.

For this reason it is gratifying to know that the Association will meet next year in Columbus. It is ten years since a meeting has occurred in southwest Georgia, and consequently the membership from that section is smaller than from any other portion of the State. It is to be hoped that the presence of the Association will act as a stimulus and that large accessions to membership will result.

The recent meeting in Augusta was a successful one. A number of valuable papers were read, and the discussions which followed were animated and instructive. The society was increased by the addition of more than fifty new members. The Association was entertained with Augusta's proverbial hospitality, and every member was made to feel that a hearty welcome was accorded him.

The remarkable increase in membership at this meeting was due to the admirable course adopted by the secretary, Dr. K. P. Moore, in sending out invitations to every regular physician in the State to be present in Augusta, and to apply for membership. Dr. Moore has worked untiringly for the interest of the Association during his term of office, and deserves the highest praise for the success which has attended his endeavors.

